# ALDH^+^ Anaplastic Thyroid Cancer Cells Show Vulnerability to a Pharmacologic Inducer of Centrosome Declustering

**DOI:** 10.1158/2767-9764.CRC-25-0807

**Published:** 2026-05-19

**Authors:** Henry Guanghao Yu, Krikor Bijian, Jie Su, Dominik Wernic, Moulay A. Alaoui-Jamali

**Affiliations:** 1 Department of Oncology, Lady Davis Institute for Medical Research of the Sir Mortimer B. Davis-Jewish General Hospital, McGill University, Montreal, Canada.; 2 Department of Medicine, Faculty of Medicine, McGill University, Montreal, Canada.

## Abstract

Excessive centrosome activation is detrimental to normal cells by hampering bipolar spindle formation during mitosis and inducing cell death. In the highly aggressive anaplastic thyroid cancer (ATC), cells expressing the aldehyde dehydrogenase–positive (ALDH^+^) stem cell trait are enriched in supernumerary centrosomes and tolerate centrosome amplification, which offers these cells a growth advantage. Our earlier work characterized the chromosomal instability (CIN) within this cell population, which was due to dysfunctional centrosomes manifesting as a deficiency in pericentriolar material. Here, we report a novel 2-4-morpholinoanilino-6-[(2-exo-norbornyl) amino]-purine (MEAP) that preferentially restores centrosome microtubule nucleation activity in centrosome-deficient ALDH^+^ ATC cells. Exposure of these cells to MEAP induced pericentriolar accumulation and microtubule nucleation activity of supernumerary centrosomes, resulting in spindle multipolarity. Consequently, MEAP was capable of preferentially eliminating ALDH^+^ ATC cell population, thus reducing cell spherogenesis and self-renewal capacity. In a preclinical ATC mouse model, MEAP preferentially eliminated ALDH^+^ cell clusters, increased the rate of spindle multipolarity, decreased CIN, and generated a robust antitumor response. Lastly, we identified that the selective activity of MEAP in ALDH^+^ cells involved the modulation of NEDD9–STAT3 signaling. Together, these findings support the potential of targeting centrosome amplification as an alternative therapeutic approach for aggressive ALDH^+^ cancers failing first-line therapeutics.

**Significance:**

This study identifies MEAP as the first selective vulnerability of ALDH^+^ ATC stem cells, their dysfunctional supernumerary centrosomes. Unlike broad chemotherapeutics, MEAP exploits this cancer-specific centrosome amplification by paradoxically hyperactivating them via NEDD9–STAT3 disruption, forcing multipolar mitosis and ALDH^+^ elimination while sparing bulk tumor cells. Validates centrosome declustering as a novel therapeutic axis for aggressive, stem-like cancers failing standard therapies, with robust *in vivo* efficacy and a mechanistic framework (NEDD9–STAT3–centrosome axis) for precision translation.

## Introduction

Tumor cell heterogeneity, a hallmark of advanced cancers, involves the intricate interactions between tumor cells and infiltrating host immune and inflammatory cell populations within the tumor tissue microenvironment. Mounting evidence across various cancer types highlighted the presence within a tumor mass of cancer cell variants expressing stem cell markers, including high aldehyde dehydrogenase 1A1 (ALDH 1A1), the focus of this study. ALDH 1A1 is an enzyme involved in embryogenesis and development, in part via regulation of retinoic acid biosynthesis. This cell subpopulation has been found to drive aggressiveness and drug resistance in multiple cancers, including anaplastic thyroid carcinomas (ATC), the most aggressive subtype of thyroid cancers. Tumor cells expressing high ALDH 1A1 (ALDH^+^) are predominant in ATC and exhibit undifferentiated features (reviewed in ref. 1). In a previous study using a panel of ATC cells and tumors, we confirmed that the ALDH 1A1–positive (ALDH^+^) ATC subpopulation express “stem-like” traits, including multipotency, sphere-forming potential, higher tumor seeding capacity, and aggressive tumorigenicity *in vivo* compared with cells expressing low ALDH 1A1 (ALDH^−^; ref. 2). In contrast to ALDH^−^ cells, we have shown that ALDH^+^ cells possess increased chromosomal instability (CIN), at least in part owing to transcriptional upregulation of the scaffolding protein NEDD9 and its downstream targets (2). We also demonstrated that ALDH^+^ cells are highly efficient at propagating CIN due to their intrinsic tolerance for both centrosome amplification and micronuclei formation. Indeed, the coexistence of centrosome abnormalities and CIN is commonly seen in several advanced cancers (3–5).

In this study, we identified a selective vulnerability of ALDH^+^ ATC cells to a pharmacologic agent inducing centrosome declustering. Compared with ALDH^−^ cells, ALDH^+^ ATC cells were hypersensitive to a novel 2,6-disubstituted purine (MEAP) derived from reversine, a purine derivative previously found to induce cell dedifferentiation (6, 7), in part via its ability to inhibit the spindle checkpoint regulators Aurora B kinase (8, 9) and MPS1, which operates downstream from Aurora B (10). We demonstrate that MEAP preferentially attenuates ATC cell stemness and induces centrosome declustering and G2/M arrest of ALDH^+^ compared with ALDH^−^ ATC cells. MEAP also exhibited potent antitumor activity *in vivo*, selectively targeting ALDH^+^ ATC cells, impairing mitosis, reducing CIN, and suppressing tumor growth.

## Materials and Methods

### Immunohistochemistry

Immunohistochemistry (IHC) was carried out as described earlier (11). Incubations with the primary antibodies diluted in PBS were conducted overnight at 4°C for anti-NEDD9 (Abcam, 1:100), anti-ALDH1A3 (Novus Biologicals, 1:250), anti-pericentrin (Abcam, 1:5,000), or anti–γ-tubulin (Abcam, 1:5,000). The sections were washed and incubated with secondary antibodies (Advanced HRP Link, DakoCytomation, K0690, Denmark) for 30 minutes, followed by the polymer detection system (Advanced HRP Link, DakoCytomation) for 30 minutes at room temperature. Reactions were developed with a solution containing 0.6 mg/mL of 3,3′-diaminobenzidine tetrahydrochloride (DAB, Sigma) and 0.01% H_2_O_2_ and then counter-stained with Mayer’s hematoxylin, dehydrated, and mounted with a glass coverslip. Positive controls (a tissue known to contain the antigen under study) were included in all reactions in accordance with manufacturer’s protocols. The negative control consisted in omitting the primary antibody and incubating slides with PBS and replacing the primary antibody with matching isotype. Quantification of γ-tubulin symmetry is based on ImageJ measurements of the relative DAB intensity of each centrosome pairs, following the same formula as for immunofluorescence measurements.

### Cell culture

THJ-11T and THJ-16T were generously provided by Dr. J. Copland (Mayo Clinic). These cell lines are derived from clinic patients with ATC and confirmed to match the primary tumor sample via DNA short tandem repeat analysis (12). 8505C cells were purchased from the American Type Culture Collection in 2015. They are maintained in RPMI 1640× with 5% FBS, 1× nonessential amino acids, 1 mmol/L sodium pyruvate, and 10 mmol/L HEPES. Microplasma was monitored by PCR (ABM G238). All cell lines were maintained in culture no longer than 3 months.

### Flow cytometry quantification of ALDH activity and sorting by ALDH status

ALDH activity was detected using ALDEFLUOR Staining Kit (StemCell Technologies). Live cells were harvested after treatment, washed once with PBS, and resuspended in ALDH assay buffer before splitting into two tubes, one with DEAB and one without. The cells were incubated in 100 μL of assay buffer + 1 μL of 300 nmol/L Aldefluor reagent and 1 mL of DEAB reagent for 15 to 40 minutes (optimized by cell line) at room temperature. The cells were then centrifuged and resuspended in new assay buffer + 5 μL of 7-AAD. 7-AAD staining is used to exclude necrotic cells during FACS analysis. The Aldefluor intensity is measured using BD FACScalibur. The samples with DEAB were measured first, followed by the matching sample without DEAB added; positivity is determined by the difference in fluorescence between +DEAB and −DEAB samples. Cell sorting by ALDH status was conducted using the same protocol in a sterile environment, using the BD-FACSAria machine. To ensure a high purification rate, we recovered only the cell population with the 20% highest or lowest Aldefluor staining respectively. Sorted cells were allowed recovery for 3 days before being reevaluated for ALDH positivity.

THJ-11T and THJ-16T were sorted based on Aldefluor activity. 8505c had basal 20% to 30% rate of ALDH^+^ cells but intensity-wise showed poor separation of the ALDH^−^ and ALDH^+^ cells and thus could not be efficiently sorted. THJ-11T had a basal 20% to 40% rate of ALDH^+^ cells and exhibited good separation of ALDH^+^ and ALDH^−^ cell population, but the ALDH^−^ cells after sorting displayed only 20% of the doubling rate compared with ALDH^+^ cells. THJ-16T had a basal 20% to 40% rate of ALDH^+^ cells, exhibited strong separation of ALDH^+^ and ALDH^−^ cell populations, and displayed relatively equal double rating between the ALDH^+^ and ALDH^−^ populations after sorting, which was optimal for fair phenotype studies following genetic or pharmacologic perturbations.

### Immunofluorescence microscopy

For immunofluorescence microscopy, cells were fixed in 4% paraformaldehyde or 100% methanol and permeabilized using 0.2% Triton-X. The centrosome, mitotic spindle, and DNA were visualized using anti-pericentrin (ab4448, Abcam, 1:2,000), anti–α-tubulin (DM1A, Millipore Sigma, 1:1,000), anti–γ-tubulin (GTU-88, Abcam, 1:1,000), anti–γ-tubulin (EPR16793, 1:1,000), and DAPI, respectively. Immunofluorescence images were obtained using a Quorum wave FX spinning disk (SD) confocal microscope or LSM800 Airyscan (Zeiss) confocal laser scanning microscope and analyzed using Volocity (PerkinElmer) or ImageJ.

To quantify spindle multipolarity in dividing cells, 60 or more random images across three biological replicas for each condition were taken at 40× or 63× magnification. At least 80 cells mitotic cells were counted per condition per replica. Representative images were captured at 63× magnification. Mitotic cells are discriminated by three criteria: chromosome condensation as visualized by DAPI, the appearance of the mitotic spindle as visualized by α-tubulin, and increased centrosome pericentriolar materials (PCM) as visualized by pericentrin (PCNT). Spindle multipolarity were identified based on the presence of >2 spindle poles radiating from distinct PCNT foci in one cell. The number of declustered centrosomes is quantified as a percentage of total mitotic cells.

To quantify centrosome amplification, cells were PCNT antibodies and visualized at 63× magnification. A cell was defined as centrosome amplified by the presence of >2 PCNT-positive dots. Images were captured using a LSM800 Airyscan (Zeiss) confocal laser scanning microscope.

For centrosome proteins intensity assays, images were captured at 63× using focusing on the middle (maximal size and intensity) of an individual centrosome. Quantification was confirmed using two methods. The first is by drawing a circle around an individual centrosome in volocity software, measuring the sum integrated pixel intensity, and then subtracting the background fluorescence (measured by placing a circle of the same dimension away from the centrosome). The second way is, in ImageJ, drawing a 2.5-μm line through the center of one or two centrosomes then using the “plot profile” function of Fiji/ImageJ software to measure the integrated area after subtracting the background. Quantification involved three biological replicates in which 15 cells were scored for each condition in each repeat. Cells with two or more than two centrosomes were scored separately. For cells with two centrosomes, only centrosome pairs less than 1 μm apart, directly adjacent to the nucleus, were included. Centrosome nucleation symmetry is calculated using the formula (peak intensity of weak centrosome/peak intensity of strong centrosome) for cells with two centrosomes or (average peak intensity of weaker 50% of the centrosomes/average peak intensity of the stronger 50% of centrosomes) in the case of centrosome amplification.

For imaging of Aldefluor activity, 1:1,000 diluted Aldefluor reagent in Aldefluor buffer was added to cells seeded in 96-well dishes, and images were captured using a fluorescent microscope as described.

### Microtubule regrowth assay

The microtubule (MT) regrowth assay was performed as previously described (13). Briefly, ATC cells were treated with nocodazole for 16 hours. Cells were then given cold medium and placed on ice for 90 minutes. Thereafter, MT regrowth was then induced by prewarmed media and placing the cells in 37°C for the indicated time (30–60 seconds), when they were subsequently fixed with 4% paraformaldehyde and stained for anti–α‐tubulin and anti–γ‐tubulin. For compound treatments, the dose reported was at the specified times prior to the induction of MT regrowth.

### Western blotting assay

Subconfluent cells were washed 3× with PBS, lysed in RIPA buffer (50 mmol/L Tris-HCl at pH 7.5, 150 mmol/L sodium chloride, 1% Triton-X-100, 0.1% SDS, 2 mmol/L EDTA, and 25 mmol/L sodium fluoride) with 1 mmol/L PMSF and protease inhibitor cocktail (Roche) on ice for 30 minutes, and centrifuged at 13,000 rpm for 20 minutes. Cell lysates was mixed with SDS buffer (Tris at pH 6.8, 20% glycerol, 5% SDS, bromophenol blue, and β-mercaptoethanol), boiled for 5 minutes, loaded into 8% to 15% SDS-PAGE gels, transferred to polyvinylidene difluoride membranes, and then blotted with the primary antibodies. The primary antibodies used were as follows: anti-NEDD9 (Cell Signaling Technology, 4044), anti–Tyr705-STAT3 (Cell Signaling Technology, 9131), anti-STAT3 (Santa Cruz Biotechnology, sc-482), anti–phospho-AURKA (Thr288)/B (Thr232)/C (Thr198; Cell Signaling Technology, 2914), anti-AURKA (Cell Signaling Technology, 14475), anti-GAPDH (Sigma-Aldrich Corporation, G9545), and anti-FLAG (Sigma-Aldrich Corporation, F1804). Secondary antibodies for Western blot assays were as follows: anti-mouse IgG peroxidase–conjugated (Bio-Rad Laboratories, 172-1011), anti-rabbit IgG peroxidase–conjugated (Bio-Rad Laboratories, 170-6515).

### Coimmunoprecipitation

ALDH^+^ THJ-16T cells were lysed in NP-40 buffer (50 mmol/L Tris-HCl pH 7.5, 150 mmol/L NaCl, 1% NP-40, 1 mmol/L EDTA, and phosphatase/protease inhibitors). Clarified lysates (500–800 μg) were incubated overnight at 4°C with 2 to 4 μg anti-NEDD9 or isotype IgG, followed by 1 hour with protein A/G agarose. Beads were washed 4× in lysis buffer, and bound proteins were eluted in SDS sample buffer, resolved by SDS–PAGE, and immunoblotted for NEDD9 and STAT3.

### Tumorsphere-forming assays

The tumorsphere medium was prepared with DMEM-F12 (3:1, Invitrogen) containing 2% B27 supplement (Invitrogen) and 20 ng/mL epidermal growth factor (Sigma-Aldrich). Tissue culture dishes were coated with a polyhydroxyethylmethacrylate polymer (polyHEMA, Sigma-Aldrich) to facilitate sphere formation. Briefly, polyHEMA was dissolved in 95% ethanol at 12% (w/v). A working solution was made by further dilution of 1:10 in 95% ethanol and was added to 24-well plates at 0.1 mL per well. A hydrophobic surface was formed after the polyHEMA solution dried out at room temperature in a tissue culture hood. Single-cell suspensions were seeded at two dilutions of 300 and 1,000 cells per well in six-well replicas. For compound-treatment, drugs were mixed with initial cell suspension. After 10 days, the number of tumorspheres (>50 μm) was microscopically counted and statically analyzed. Spheres were quantified using both seeding dilutions where possible, though dilutions in which too few (<5) or too many (>50) spheres were not used for quantification. Representative images are generated from the same seeding concentration.

### Clonogenic and cell proliferation assays

Wild-type (WT) or stable cell lines were harvested from exponential-phase cultures, counted, and plated at the described density in 12-well plates for clonogenic assay and 96-well plates for MTT (Corning Costar). Four hours after plating, the compounds or vehicle were delivered once at the mentioned concentration. Clonogenic assay: after 5 days of incubation in the presence of the compounds, the cells were stained with 0.5% crystal violet and 6% glutaraldehyde, and colonies were counted using GelCount Tumor Colony Counter from Oxford Optronix, Ltd. Each experiment was done in biological triplicates, and each clonogenic graph was constructed from at least two independent experiments. MTT: after 4 days of incubation in the presence of the compounds, 20 μL MTT solution (5 mg/mL MTT in PBS, filter-sterilized) was added to each well and incubated in a 37°C incubator for 4 hours, after which the medium was carefully removed by pipetting and the MTT formazan was resuspended using DMSO. Absorbance was measuring using a spectrophotometric plate reader at 550 nm.

### *In vivo* anticancer activity

Animal experiments were performed in compliance with institutional and federal guidelines after approval from the McGill University Facility Animal Care Committee (protocol #4515). NOD-SCID mice were purchased from Charles River Laboratories (St. Zotique). Orthotopic tumor model: 6- to 8-week-old NOD-SCID female mice were anesthetized (90 mg/mL ketamine and 10 mg/mL xylazine). A horizontal incision was made to cut the skin, and subcutaneous tissues to expose the central component of the neck and the overlying strap muscles were dissected away from the right thyroid. Cells (5 × 10^5^ 8505c) in 10 μL of serum-free RPMI medium were injected into the right thyroid gland. Following injection, the incision was closed using 3-0 nylon sutures. Mice were monitored daily for postsurgical complications. Once tumor became palpable on neck examination, mice were treated with three doses of MEAP (30 mg/kg) or Taxol (5 mg/kg) and then sacrificed a week after the last treatment to evaluate for ALDH1A3-positive staining by IHC (*n* = 3–5). Subcutaneous tumor model: ATC cells were injected subcutaneously into the right flank of NOD-SCID mice. Once tumor volume reached 40 to 80 mm^3^, mice were given triweekly injection of vehicle, MEAP (30 mg/kg), or Taxol (5 mg/kg), and tumor size/body weight were recorded every 3 days. No treatment-related changes in body weight, food or water consumption, or behavior were observed during the observation period (*n* = 8). Limiting dilution transplantation: tumor-initiating cell frequency was determined using extreme limiting dilution analysis (ELDA) with a Poisson–stochastic model assuming a single tumor-initiating event per mouse. Data were analyzed using the statmod package (v1.5.1) in R v4.4.1.

### Cell-cycle analysis

Cells were pulse-labeled with 10 μmol/L BrDU for 1 hour, fixed in cold 70% ethanol for 2 hours, and then rehydrated in PBS. Cells were thereafter treated with 2N HCl for 10 minutes, followed by 0.1 mol/L Na2B4O7 for 5 minutes, and then labeled with Alexa 647 anti-Brdu (BioLegend) and DAPI. For flow cytometry acquisition, cells were gated based on FSC-A/SSC-A, FSC-H/FSC-A, and DAPI-H/DAPI-A to discriminate 10 cells.

### Chemistry

The 2-4-morpholinoanilino-6-[(2-exo-norbornyl)amino]-purine (MEAP) was synthesized as previously described (14). Briefly, MEAP as a racemic mixture, was obtained from the reaction of exo-2-aminonorbornane (racemic, CAS: 7242-92-4, Sigma-Aldrich, cat. #179604) with 6-chloro-2-fluoropurine (CAS:1651-29-2, Oakwood Chemical, cat. #009088) in n-butanol at 100°C for 16 hours during the first synthetic step of this method. The structure/purity of all analogs was confirmed by ^1^H NMR, ^13^C NMR, and HRMS. Other compounds were purchased: XL-019 (MedKoo Biosciences), VX-680 (Cayman Chemical), PF-573228 (Tocris Bioscience), and reversine (Sigma-Aldrich). Vandetanib (Selleckchem), cabozantinib (Selleckchem), RO4929097 (Selleckchem), U0126 (Tocris Bioscience), PP2 (ADOOQ), and IWP-2 (Tocris Bioscience) were used.

### Statistical analysis

Data shown as the mean ± SD (*n* = biological replicates). Two groups: unpaired two-tailed *t* test (Welch if unequal variance). ≥3 groups/time: one-way ANOVA (Tukey HSD *post hoc*). Multiple comparisons: two-way ANOVA (Sidak *post hoc*). Mitotic/spindle: *χ*^2^ (proportions). TIC frequency: ELDA (R statmod). Prism 10/GraphPad.

## Results

### MEAP induces centrosome MT nucleation in ATC cells

We evaluated the impact of reversine, a 2,6-disubstituted purine known to induce cell dedifferentiation by inhibiting AuroraB/MPS1 kinases (6–10, 13) and a series of its analogs on the proliferation and colony forming abilities of ATC cells, THJ-11T and THJ-16T. Among these, MEAP showed highest potency in inhibiting ATC growth (Supplementary Table S1). *In vitro* kinase profiling (Supplementary Fig. S1A) showed that MEAP exhibits multikinase activity, inhibiting all three Aurora isoforms (AURKA, AURKB, and AURKC) as well as several NEDD9-associated kinases, including FAK, with greater potency than reversine. Consistent with these biochemical data, short-term MEAP exposure reduced phosphorylation of Aurora-A and FAK in ALDH^+^ THJ-16T cells in a dose-dependent manner (Supplementary Fig. S1B). Because NEDD9 functionally couples to Aurora kinases and FAK at centrosomes and focal adhesions, respectively (15, 16), these results support the use of MEAP as a tool to interrogate NEDD9-dependent signaling in ATC.

**Figure 1.**
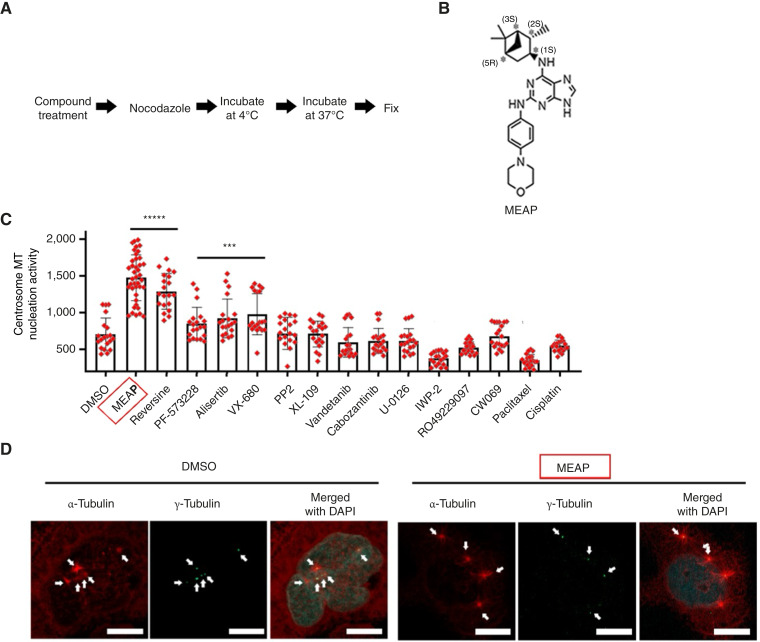
Identification of MEAP as an inducer of centrosome-mediated MT nucleation in ATC cells with poorly nucleated supernumerary centrosomes. **A,** Experimental scheme of the MT regrowth assay. **B,** Chemical structure of MEAP. **C,** Quantification of relative centrosome MT nucleation activity in compound-treated THJ-16T cells based on α-tubulin staining intensity. At least 25 centrosomes were scored per experiment, *n* = 3 biological replicates. **D,** Representative images of MT regrowth in DMSO- or MEAP-treated THJ-16T cells with supernumerary centrosomes. Note that DMSO-treated cells exhibit supernumerary centrosomes with poor MT nucleation capacity, whereas MEAP-treated cells show robust MT nucleation at all supernumerary centrosomes. Scale bars, 10 μm. Data representation (mean ± SD). ***, *P* < 0.001; *****, *P* < 0.00001 by one-way ANOVA with Tukey *post hoc* test (Global *P* < 0.001). MEAP showed significantly higher centrosome MT nucleation activity than reversine (*P* < 0.01) and all other compounds tested.

To test whether MEAP modulates centrosome function, a vulnerability in ALDH^+^ ATC cells with supernumerary centrosomes (2), we assessed centrosome-mediated MT nucleation using a standard MT regrowth assay (13, 17, 18) following nocodazole and cold-induced MT depolymerization. In this assay, MEAP exhibited significantly enhanced activation of centrosomal MT growth compared with several known inhibitors of centrosome function and stem cell signaling, including inhibitors of AURKA (alisertib), FAK (PF-573228), SRC (PP2), JAK2 (XL019), and MEK1/2 (U0126); an HSET inhibitor known to disrupt centrosome clustering (CW069; ref. 19); the Wnt signaling inhibitor IWP-2 (20); and two nonselective chemotherapeutic agents, cisplatin and paclitaxel. In parallel, we compared MEAP with clinically used multitarget receptor tyrosine kinase (RTK) inhibitors vandetanib (21) and cabozantinib (22), which primarily target VEGFR, EGFR, MET, and RET, as well as with the γ-secretase–dependent Notch pathway inhibitor RO4929097 (Fig. 1A–C; ref. 23). Importantly, MEAP-induced centrosome MT nucleation was also observed in cells with supernumerary centrosomes (Fig. 1D), underscoring its activity in ATC cells exhibiting centrosome amplification. Collectively, these findings identify MEAP as a potent inducer of centrosome-mediated MT nucleation in ATC cells.

### MEAP increases PCM accumulation at inactive centrosomes to enhance centrosome nucleation capacity

A key determinant of centrosome MT nucleation potential during interphase is the amount of PCM, such as PCNT, which supports MT polymer formation (24–28). To evaluate PCM accumulation across different stages of the centrosome cycle, we costained for centrin-1 (a centriole marker) and PCNT and quantified MEAP-induced changes in PCM levels via fluorescence intensity.

**Figure 2.**
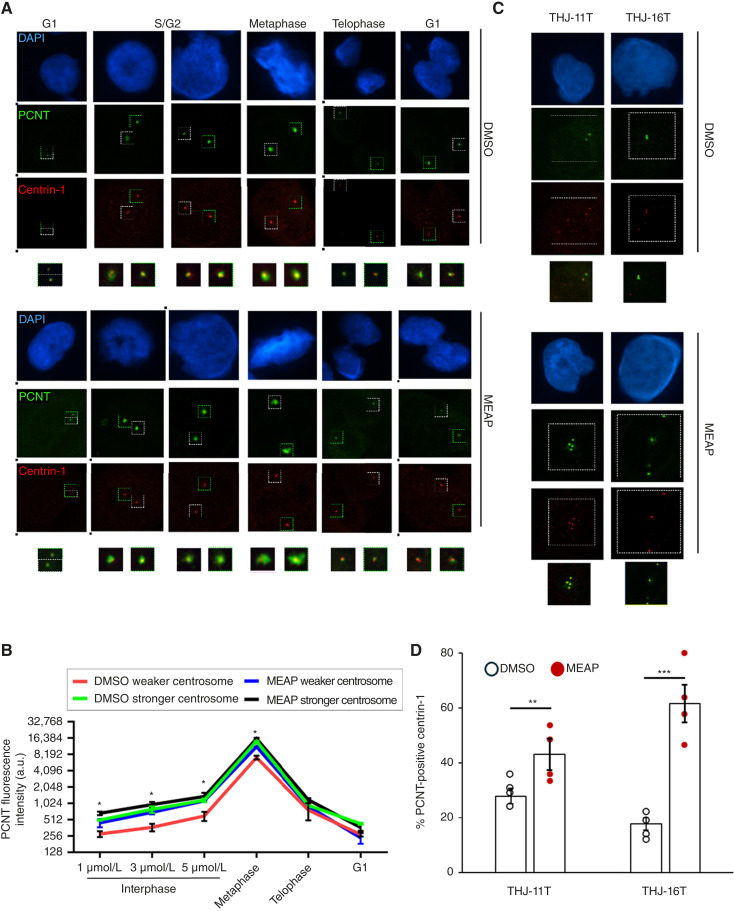
MEAP induces PCM accumulation at inactivate centrosomes during interphase. **A,** Representative images of centrin-1 (centriole marker) and PCNT costaining in THJ-16T cells with a normal number of centrosomes. In DMSO-treated cells, one of the two centrosomes often displays reduced PCNT staining during interphase, whereas MEAP-treated cells show more uniform PCNT accumulation across both centrosomes. Cell-cycle stage was inferred based on centriole duplication status (before duplication in first and last columns) and the distance between centrosomes, ensuring equivalent comparisons between DMSO- and MEAP-treated cells. **B,** Quantification of PCNT fluorescence intensity at individual centrosomes reveals that MEAP primarily increases PCNT levels at the less active (“weaker”) centrosome of the pair. Centrosome identity was determined by intercentrosomal distance during interphase. Quantification was performed in Fiji/ImageJ by drawing a 2.5-μm line through the center of one or both centrosomes and using the “Plot Profile” function to calculate integrated intensity after background subtraction. Data represent three biological replicates, with 25 cells scored per condition per replicate (*n* = 3). Data representation (mean ± SD) *, *P* < 0.01 by one-way ANOVA with Tukey *post hoc* test (weak DMSO vs. weak MEAP). **C,** Representative images of centrin-1 and PCNT costaining in THJ-11T and THJ-16T cells with supernumerary centrosomes. DMSO-treated cells show frequent PCNT-deficient centrioles, whereas MEAP-treated cells exhibit widespread PCNT acquisition. **D,** Quantification of the percentage of centrioles with visible PCNT accumulation. MEAP significantly increases the proportion of PCM-positive centrioles in both cell lines. At least 25 cells with centrosome amplification were analyzed per condition across three biological replicates (*n* = 3). Data representation (mean ± SD) **, *P* < 0.01; ***, *P* < 0.001 by *t* test with Welch correction.

We first examined MEAP’s effects in ATC cells with a normal number of centrosomes. By controlling for centriole replication status and intercentriolar distance to identify centrosome cycle stages, we found that MEAP significantly increased PCNT and γ-tubulin levels following centriole disengagement (two singlet centrioles), with this enhancement continuing through centrosome maturation (G1 to G2) and into early mitosis. Notably, MEAP had no effect on PCM levels during telophase, suggesting that it does not influence PCM shedding during mitotic exit (Fig. 2A and B).

Next, we assessed MEAP’s effect on PCM accumulation at supernumerary centrosomes. Specifically, we quantified the proportion of centrioles that recruited PCNT during interphase, given that PCM-deficient centrioles are a characteristic feature of ALDH^+^ ATC cells. MEAP treatment increased the frequency of PCM-positive interphase centrioles from 23% ± 4% to 46% ± 9% in THJ-11T cells and from 18% ± 4% to 52% ± 6% in THJ-16T cells (Fig. 2C and D). These results indicate that MEAP promotes PCM recruitment to centrosomes, thereby enhancing the activation and nucleation capacity of supernumerary centrosomes during interphase.

### MEAP selectively induces spindle multipolarity and G2/M phase arrest in ALDH^+^ ATC cells

One of the most common causes of cell death associated with centrosome amplification is mitotic catastrophe due to unresolved spindle multipolarity (29, 30). As the spindle-forming potential of centrosomes depends on PCM accumulation during interphase, MEAP would be expected to enhance multipolar spindle formation during mitosis.

**Figure 3.**
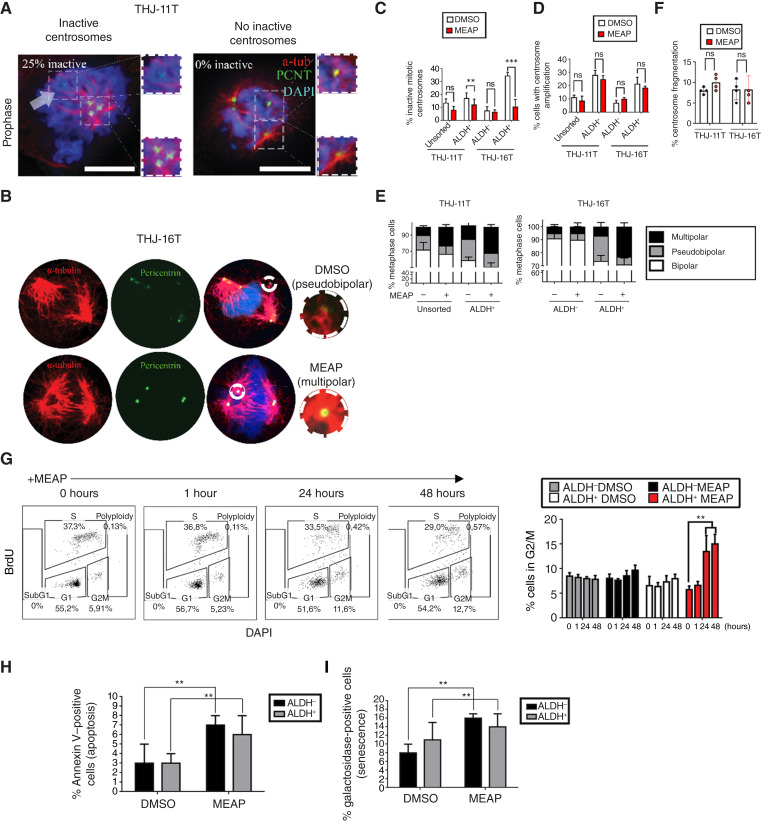
MEAP preferentially induces multipolar spindles and G2/M arrest in ALDH^+^ ATC cells. **A,** Representative image of “inactive” centrosomes observed in ALDH^+^ cells at mitosis, defined as centrosomes which are visibly devoid of spindle-forming capacity. Cell model represented is THJ-11T. **B,** Representative images of a pseudobipolar and multipolar spindle in THJ-16T cells with supernumerary centrosomes. **C,** Quantification of % inactivated centrosomes during mitosis. Sorted cells were treated with DMSO or 300 nmol/L MEAP for 48 hours. **D,** Quantification of the ratio of cells with supernumerary centrosomes (>2 centrosomes). Sorted cells were treated with DMSO or 300 nmol/L MEAP for 48 hours. **E,** Quantification of spindle configuration in ALDH^+^ cells treated with DMSO or MEAP. Pseudobipolar spindles are defined as >2 centrosomes assembled into two spindle poles, and multipolar spindles were defined as >2 centrosomes assembled into >2 spindle poles (represented in **B**). Spindle multipolarity THJ-11T/16T (MEAP): ALDH^+^ 32 ± 5% vs. 8% ± 3% ALDH^−^ (*P* < 0.001, unpaired *t* test, *n* = 3), and no change ALDH^−^ (*P* = 0.42). **F,** Impact of MEAP on centrosome fragmentation (defined as % of PCNT-positive structures lacking centrin-1). **G,** Representative flow cytometry panel and quantification of BrdU/DAPI cell-cycle compartmentalization assay of sorted THJ-16T cells treated with 300 nmol/L MEAP or DMSO. Treated cells were harvested at the indicated time points. *n* = 3 biological replicates. **H,** Quantification of propidium iodide (PI)/Annexin V apoptosis assay of sorted THJ-16T cells treated with MEAP. *n* = 3 biological replicates. **I,** Quantification of β-galactosidase senescence assay of sorted THJ-16T cells treated with MEAP. *n* = 3 biological replicates. Scale bars, 10 μm. Data representation (mean ± SD) **, *P* < 0.01; ***, *P* < 0.001 by one-way ANOVA with Tukey *post hoc* test.

We first quantified centrosome spindle-forming capacity in DMSO- and MEAP-treated THJ-11T and THJ-16T cells. Indeed, approximately one thirds of centrosomes in DMSO-treated cells exhibited minimal or no spindle-forming capacity (Fig. 3A). MEAP treatment significantly increased the percentage of centrosomes capable of generating robust mitotic spindles, thereby intensifying spindle multipolarity (Fig. 3B and C). Importantly, MEAP did not alter the overall frequency of centrosome amplification (Fig. 3D).

MEAP also shifted the ratio of spindle configurations by increasing multipolar spindles and decreasing pseudobipolar spindles in both cell lines after 48 hours at 300 nmol/L (Fig. 3B and E). Successful production of two viable daughter cells after multipolar spindle formation generally requires the assembly of extra spindle poles into a “pseudobipolar” configuration (30–32). Prior work has shown that elevated PCM accumulation during interphase disrupts this pseudobipolar adaptation (13). This suggests that MEAP enhances the spindle-forming activity of supernumerary centrosomes, thereby impairing the ability to establish pseudobipolar spindles. Supporting this, cells with pseudobipolar spindles typically contained nonpolarized, spindle-deficient centrosomes, whereas cells with multipolar spindles had only robust spindle-forming centrosomes (Fig. 3B). The multipolar spindles in MEAP-treated cells appeared structurally normal (Fig. 3B), and MEAP had no significant effect on non–centrin-1–associated PCNT levels, suggesting that spindle multipolarity was not due to PCM fragmentation (Fig. 3F).

Inactive supernumerary centrosomes were disproportionately prevalent in ALDH^+^ cells (Fig. 3B and C), indicating that this population would be more sensitive to MEAP-induced centrosome dysfunction. Indeed, MEAP induced significantly greater spindle multipolarity in ALDH^+^ compared with ALDH^−^ cells (Fig. 3E). Cell-cycle analysis showed that MEAP treatment led to G2/M accumulation in synchronized ALDH^+^ but not ALDH^−^ cells (Fig. 3G), consistent with selective mitotic disruption.

In contrast, MEAP induced similar levels of apoptosis (Fig. 3H) and senescence (Fig. 3I) in both populations, indicating that whereas spindle multipolarity and G2/M arrest were selective for ALDH^+^ cells, the ultimate fate of these arrested cells cannot be definitely assigned.

### MEAP selectively eliminated ALDH^+^ cells and suppressed ATC spherogenesis

ALDH^+^ ATC cells exhibit elevated stemness-associated features, including self-renewal (e.g., tumorsphere-forming capacity) and tumor-initiating potential, compared with the bulk tumor population (2, 33–35). Given that MEAP preferentially induces spindle multipolarity and G2/M phase arrest in ALDH^+^ cells, we next investigated whether it could selectively deplete this subpopulation across multiple ATC cell lines.

**Figure 4.**
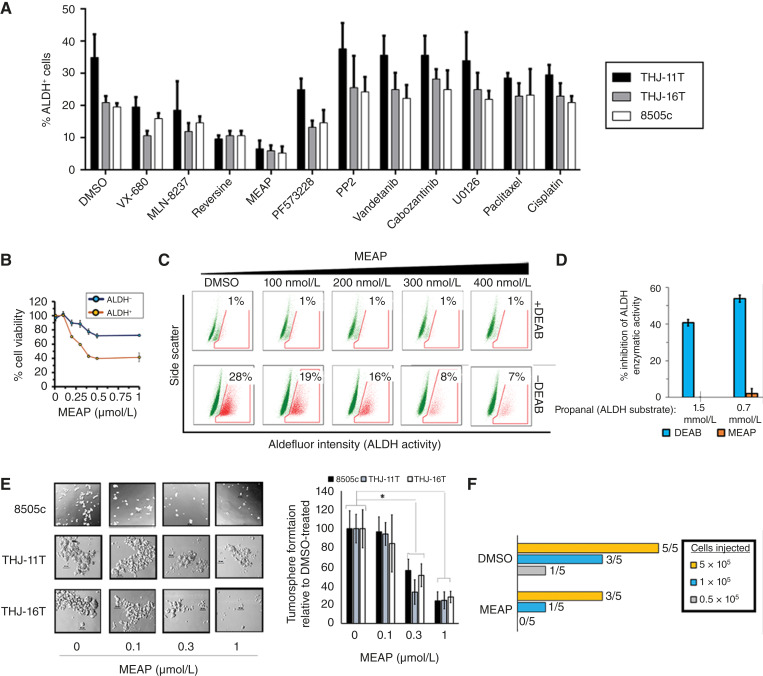
MEAP selectively eliminates ALDH^+^ ATC cells and attenuates spherogenesis and tumorigenic potential. **A,** Quantification of the impact of small molecules (300 nmol/L) on the ratio (%) of cells expressing the cancer stem-like cell marker ALDH^+^ in three ATC models. **B,** Viability assay illustrating that MEAP’s selectivity against ALDH^+^ THJ-16T cells are due to differential effects on ALDH^+^ vs. ALDH^−^ cell growth. **C,** Dose-responsive inhibition of the %ALDH^+^ cell population in THJ-16T ATC cells after 72 hours treatment of MEAP, in which the addition of the ALDH enzymatic inhibitor “DEAB” is used to establish background levels. **D,** Impact of MEAP on ALDH enzymatic assay. Propanal is added to a cell mixture together with MEAP, DEAB, or DMSO. Reaction inhibition is measured by total NAD+ reduction in MEAP- or DEAB-treated (positive control) samples compared with those treated with DMSO (negative control); decreasing dose of substrate is for assessing competitive inhibition (*n* = 3). **E,** Representative image and quantification of the impact of MEAP on tumorsphere formation in several ATC cell lines. A total of 1,000 cells were seeded in polyhema-coated plates in tumorsphere growing media (DMEM/F12 + EGF + B27). Images were taken after 7 days, using 10× magnification; for quantification, brightfield microscopy was used to count spheres more than 50 μm. Quantification is normalized to DMSO-treated for each cell line. **F,** Limiting dilution transplantation assay to measure the impact of MEAP pretreatment on ATC cell tumor seeding potential *in vivo*. THJ-16T cells were recovered for 5 days after pretreatment and injected at 5 × 10^5^, 1 × 10^5^, and 0.5 × 10^5^ cells per mouse (*n* = 5 per dose) into the right flank of NOD-SCID mice. Tumor growth was assessed by palpitation and confirmed at necropsy after 35 days. ELDA stem cell frequencies were DMSO 1/262,000 vs. MEAP 1/1,310k (*P* = 0.015); equivalent to ∼80% drop in CIC number. Data representation (mean ± SD) *, *P* < 0.01 by one-way ANOVA with Tukey *post hoc* test.

After 96 hours of MEAP treatment, the percentage of ALDH^+^ cells decreased by more than 80% in THJ-11T, THJ-16T, and 8505c cells compared with DMSO controls. A similar reduction was partially recapitulated using Aurora-A and FAK inhibitors, whereas inhibitors targeting SRC, ERK, or other tyrosine kinases, as well as chemotherapeutic agents such as cisplatin and paclitaxel, had little to no effect or slightly increased the proportion of ALDH^+^ cells (Fig. 4A). We confirmed that MEAP’s selective elimination of ALDH^+^ cells involves differential effects on cell growth (Fig. 4B), with a clear dose-dependent response (Fig. 4C), without having direct inhibition of ALDH enzymatic activity as a mechanism (Fig. 4D).

To evaluate whether MEAP’s selective targeting of ALDH^+^ cells also impaired stemness, we pretreated ATC cells with MEAP for 96 hours, allowed a 1-week recovery period, and then assessed the tumorsphere-forming and tumor-initiating capacity of the surviving cells. MEAP significantly and dose-dependently reduced tumorsphere formation in THJ-11T, THJ-16T, and 8505c cells (Fig. 4E). In a limiting dilution transplantation assay using pretreated THJ-16T cells, 1 × 10^5^ DMSO-treated cells formed tumors in three of five mice, whereas 5 × 10^5^ MEAP-treated cells were required to achieve the same tumorigenesis rate. This trend held across multiple injection cell counts, suggesting an estimated ∼80% reduction in tumorigenic potential (Fig. 4F).

Collectively, these findings demonstrate that MEAP selectively targets ALDH^+^ ATC cells and effectively attenuates ATC stemness and tumorigenicity.

### MEAP selectively eliminates ALDH^+^ cells, induces spindle multipolarity, and inhibits CIN *in vivo*

**Figure 5.**
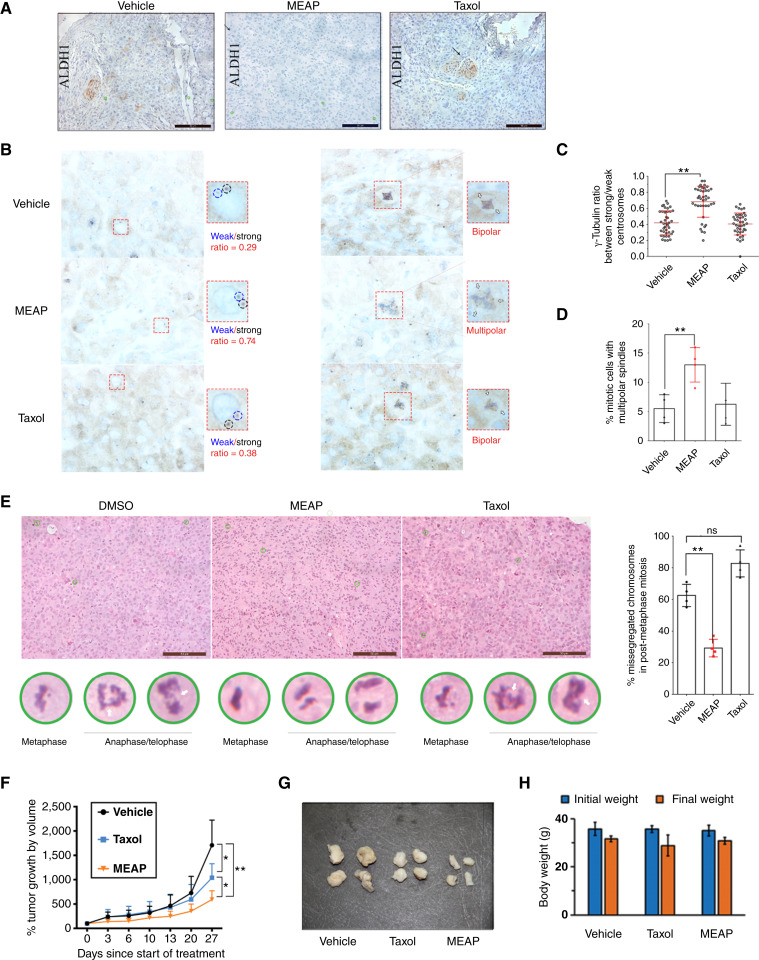
MEAP eliminates ALDH^+^ ATC cells, increases PCM levels, induces spindle multipolarity, and reduces CIN *in vivo*. **A–E,** Orthotopic model; 5 × 10^5^ 8505c cells were injected into the right thyroid of NOD-SCID mice on day 0; mice were given 3 dosages of treated DMSO (vehicle), Taxol (5 mg/kg), or MEAP (30 mg/kg; *n* = 3–5) on day 15, 17, and 20 (i.p.); mice were sacrificed, and necropsy was performed on day 30. ALDH1A3 appeared as localized clusters in DMSO/Taxol-treated tumors which are absent in MEAP-treated tumors. **A,** Brightfield images IHC staining of ALDH1A3 staining in primary thyroid tumor. **B,** IHC staining of γ-tubulin in 8505c xenografts treated with DMSO, Taxol (5 mg/kg), or MEAP (30 mg/kg). **C,** Symmetry quantification, 10 centrosome pairs from each mouse was assessed. Symmetry is measured by comparing the relative intensity of γ-tubulin staining between centrosome pairs (intensity of weak/strong). To ensure cell-cycle parity, only centrosomes of equal distance are measured. **D,** Spindle multipolarity quantification. For spindle multipolarity, at least 50 mitotic cells from each mouse were evaluated (*n* = 4). **E,** Left, representative images representing chromosomal missegregation in 8505c xenografts, observed by hematoxylin and eosin (H&E) staining. Right, missegregated chromosome quantification. Based on H&E images (10 random field per mice, *n* = 4 for each group), quantification was done for the rate of mitotic cells exhibiting chromosome missegregation (lagging chromosomes or chromosome bridges). At least 50 mitotic cells from each mouse were evaluated (*n* = 4). **F–H,** Subcutaneous model; 1 × 10^6^ 8505c cells were implanted subcutaneously into NOD-SCID mice. Treatment started once tumor reached 40–80 mm^3^, and animals were treated three times a week (i.p.) with DMSO, Taxol (5 mg/kg), or MEAP (30 mg/kg; *n* = 8). **F,** Tumor size change curve of subcutaneous growth. **G,** Representative images of subcutaneous tumors were taken after necropsy. **H,** Initial and final body weights of animals from subcutaneous model. Scale bars, 10 μm. Data representation (mean ± SD) *, *P* < 0.05; **, *P* < 0.01 by one-way ANOVA with Tukey *post hoc* test.

To evaluate the preclinical efficacy of MEAP, we used an orthotopic ATC model in which 8505c cells were inoculated into the right thyroid lobe of NOD-SCID mice on day 0. Mice were treated intraperitoneally with either vehicle (DMSO), Taxol (5 mg/kg), or MEAP (30 mg/kg) on days 15, 17, and 20 (*n* = 3–5 per group), and necropsy was performed on day 30. We selected the 8505c cell line for its higher tumor take rate (100% vs. 75% for THJ-16T; ref. 36). IHC analysis revealed the presence of abundant ALDH1-positive cell clusters in both vehicle- and Taxol-treated tumors, which were absent in MEAP-treated tumors, indicating that MEAP’s selective targeting of ALDH^+^ cells is recapitulated *in vivo* (Fig. 5A). γ-Tubulin staining demonstrated that MEAP reduced centrosome asymmetry, consistent with *in vitro* findings (Fig. 5B and C). MEAP also significantly increased the frequency of multipolar spindles relative to DMSO- and Taxol-treated groups (Fig. 5B and D), again mirroring its *in vitro* activity. Furthermore, hematoxylin and eosin staining revealed a reduction in chromosomal missegregation events during anaphase/telophase in MEAP-treated tumors (Fig. 5E). As CIN in centrosome-amplified cells is often driven by defective mitosis, this supports the conclusion that MEAP promotes mitotic catastrophe by disrupting mitosis in ATC cells with centrosome amplification (37). Finally, to assess whether MEAP could impair the progression of established tumors, we used a subcutaneous ATC xenograft model, in which consistent growth kinetics can be followed over time, in contrast to the orthotopic model. Once tumors became palpable, mice (*n* = 8 per group) were treated intraperitoneally with vehicle, MEAP (30 mg/kg), or Taxol (5 mg/kg) three times weekly for 4 weeks. MEAP treatment resulted in a ∼4-fold reduction in tumor volume compared with controls (Fig. 5F–G), without detectable toxicity or bodyweight loss (Fig. 5H). Notably, MEAP outperformed Taxol in suppressing ATC tumor growth.

Altogether, these findings demonstrate that MEAP exerts potent antitumor activity *in vivo*, selectively targets ALDH^+^ ATC cells, disrupts mitosis, reduces CIN, and inhibits tumor progression, all without apparent host toxicity in the preclinical setting.

### MEAP selectivity toward ALDH^+^ cells involves modulation of NEDD9-dependent STAT3 signaling

**Figure 6.**
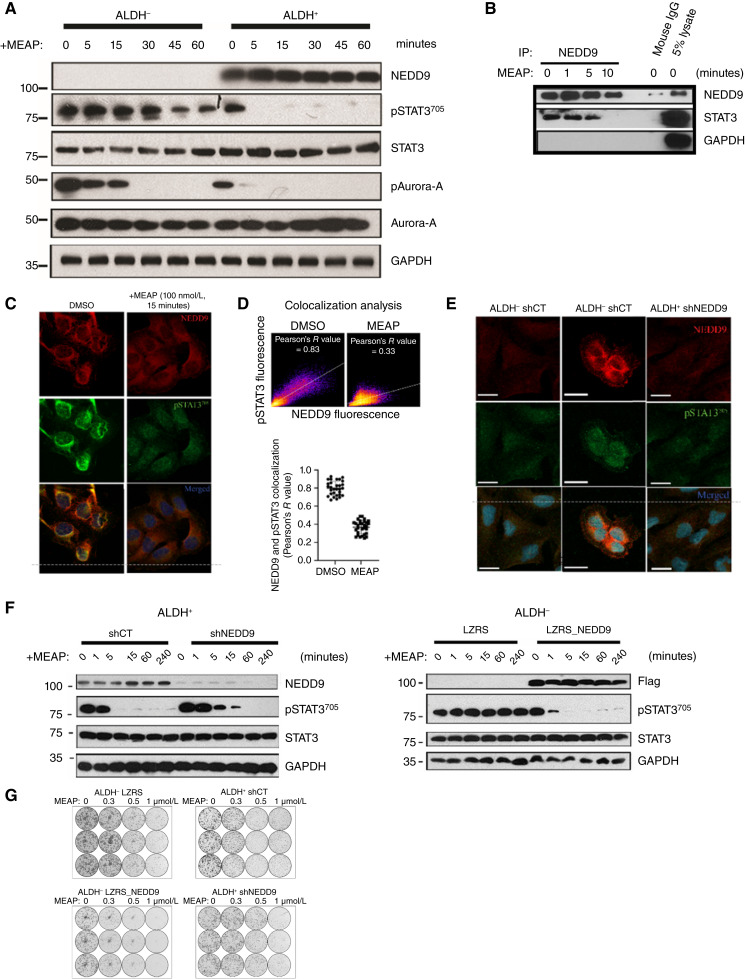
MEAP induces rapid STAT3 dephosphorylation in a NEDD9-dependent manner. **A,** Western blot illustrating the rapid kinetics at which MEAP selectively induces STAT3 dephosphorylation in THJ-16T ALDH^+^ cells. By comparison, MEAP inhibited Aurora-A nonselectively between ALDH^+^ and ALDH^−^ cells. **B,** Immunoprecipitation experiment using anti-NEDD9. THJ16T ALDH^+^ cells were treated with 100 nmol/L MEAP treatment for the indicated duration and then harvested. **C,** Immunofluorescence images illustrating the impact of MEAP on pSTAT3^705^ and NEDD9 cellular distribution. **D,** Representative panel and quantification of NEDD9/pSTAT3^705^ colocalization in ALDH^+^ cells treated with DMSO or MEAP. **E,** Immunofluorescent images of ALDH^−^, ALDH^+^, and ALDH^+^ shNEDD9 cells stained for NEDD9 and pSTAT3^705^. **F,** Immunoblots illustrating MEAP’s ability to inhibit pSTAT3-705 in THJ-16T is dependent on NEDD9 expression. ALDH^+^ and ALDH^−^ sorted cells were NEDD9-depleted or NEDD9-overexpressed and then treated with 100 nmol/L MEAP over multiple time points. (LZRS = LZRS_IRESGFP control, LZRS_NEDD9 = full-length NEDD9 cloned into LZRS-IRESGFP expression vector). **G,** Representative image of MEAP’s impact on colony formation in THJ-16T ALDH^+^ and ALDH^−^ cells with stable NEDD9 depletion or NEDD9 overexpression, respectively (LZRS = LZRS_IRESGFP control, LZRS_NEDD9 = full-length NEDD9 cloned into LZRS-IRESGFP expression vector). Cells were seeded at 1,000 cells per well and grown for 5 days (*n* = 3). Scale bars, 10 μm.

Analysis of multiple signaling events previously found to be regulated by the parental molecule reversine revealed that STAT3 signaling is the major target of MEAP in ALDH^+^ cells. Specifically, the STAT3 tyrosine phosphorylation site Y705 (pSTAT3-Y705) was rapidly depleted in ALDH^+^ cells following MEAP treatment, whereas pSTAT3 levels remained unchanged in ALDH^−^ cells (Fig. 6A). In contrast, phosphorylation of Aurora-A, previously reported to be regulated by reversine, was not affected, supporting that MEAP’s selectivity toward STAT3 signaling is restricted to the ALDH^+^ subpopulation. As we have previously found that the scaffolding protein NEDD9 is highly overexpressed in ALDH^+^ cells and that STAT3 is known as a downstream effectors of NEDD9 (2, 38), we decided to investigate whether NEDD9 and its interactome can distinguish STAT3 signaling between the ALDH± cell populations in ATC. In fact, coimmunoprecipitation of NEDD9 resulted in the pulling-down of STAT3, offering evidence of their interaction (Fig. 6B). This interaction was inhibited within 5 to 10 minutes of MEAP exposure, consistent with the timeline of STAT3 dephosphorylation. We then visualized NEDD9 and pSTAT3-Y705 intracellular expression using immunofluorescence staining. ALDH^+^ cells showed intense cytoplasmic NEDD9 and pSTAT3-Y705 staining confined to matching regions (Fig. 6C and D). Aside from the cytoplasmic regions, smaller levels of pSTAT3-Y705 and NEDD9 were observed in the nucleus and cell membrane, respectively (Fig. 6C). The addition of MEAP caused a visible decrease in pSTAT3-Y705 staining in the cytoplasm but not the nucleus (Fig. 6C and D). MEAP also caused even NEDD9 redistribution from perinuclear region (Fig. 6C). Notably, when untreated, NEDD9/pSTAT3-Y705 were generally colocalized and shared a strong linear relationship in their staining intensity, suggestive of direct interaction (Fig. 6D).

Given that disruption of NEDD9/STAT3 interaction and decrease in pSTAT3-Y705 phosphorylation appear to occur simultaneously within minutes of MEAP treatment (Fig. 6A and B), this proposed that NEDD9 was likely required for MEAP-mediated STAT3 dephosphorylation. To test NEDD9 requirement, we examined intracellular localization (Fig. 6E) and STAT3 phosphorylation kinetics (Fig. 6F) following short hairpin RNA (shRNA)-mediated NEDD9 protein knockdown with or without MEAP exposure. In fact, in ALDH^−^ cells in which MEAP treatment normally did not result in pSTAT3^705^ inhibition, ectopic overexpression of NEDD9 via LZRS expression vector was sufficient to sensitize pSTAT3^705^ levels to MEAP treatment (Fig. 6F). These findings collectively indicate that NEDD9 overexpression in ALDH^+^ cells unexpectedly sensitizes these cells to MEAP-mediated pSTAT3^705^ inhibition. As with MEAP-treatment, shNEDD9 resulted in sustained pSTAT3^705^ decrease (Fig. 6E). When treating ALDH^−^ cells stably expressing NEDD9 or ALDH^+^ cells with NEDD9 knockdown (shNEDD9R), we found that NEDD9 expression levels determined MEAP’s ability to inhibit STAT3 phosphorylation (Fig. 6F). NEDD9 expression also appeared to dictate MEAP’s overall antiproliferative effects, reinforcing the conclusion that MEAP suppresses ATC growth (Fig. 6G), at least in part, through inhibition of pSTAT3-Y705.

Together, these findings demonstrate that MEAP selectively disrupts the NEDD9–STAT3 signaling pathway in ALDH^+^ cells, leading to aberrant centrosome activation. This NEDD9–STAT3 specificity underlies MEAP’s selective targeting of ALDH^+^ cells via centrosome declustering and mitotic failure (Figs. 3F, G, and 4A), despite nonselective apoptosis/senescence (Fig. 3H and I).

### MEAP induces multipolar spindle formation and mitotic failure in centrosome-amplified ALDH^+^ cells, thereby reducing chromosomal missegregation via the NEDD9–STAT3 signaling axis

**Figure 7.**
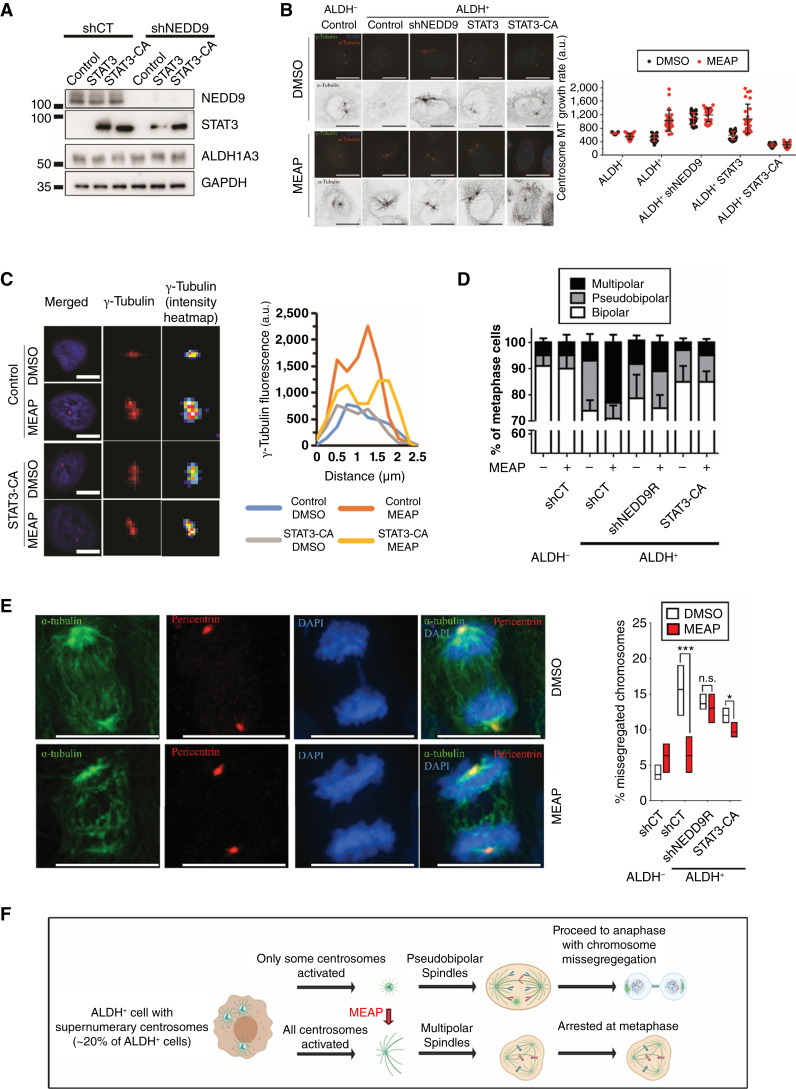
MEAP causes multipolar spindles and mitotic failure in ALDH^+^ cells harboring centrosome amplification and leads to reduced missegregated chromosomes in a NEDD9/STAT3-dependent manner. **A,** Western blot illustrating NEDD9 knockdown in combination with STAT3 or STAT3-CA (constitutively activated mutant with A662C, N664C point mutations) expression. **B,** MT regrowth assay illustrating that STAT3-CA but not STAT3 overexpression can abrogate MEAP-induced centrosome MT-nucleation. At least 25 centrosomes were scored per experiment, *n* = 3 biological replicates. **C,** MEAP induced γ-tubulin accumulation was inhibited by ectopic expression of constitutively activated STAT3 (STAT3-CA). Left, representative images and quantification of γ-tubulin levels in DMSO- or MEAP-treated (100 nmol/L, 24 hours) control or STAT3-CA–overexpressing ALDH^+^ THJ-16T cells. Right, line graph panel indicating the intensity of centrosome pairs or individual centrosomes of cells, measured using the “plot profile” function of Fiji/ImageJ software. The region quantified is depicted by a white line in the left. **D,** Quantification of the relative rate of bipolar, pseudobipolar (defined as >2 centrosomes assembled into two spindle poles), or multipolar (defined as >2 centrosomes assembled into >2 spindle poles) centrosomes in the different groups. **E,** Representative image (original magnification 63×) and quantification of the impact of MEAP on rate of missegregated chromosomes (defined by presence of lagging chromosomes or chromosome bridges). Data representation (mean ± SD) *, *P* < 0.05; ***, *P* < 0.001 by one-way ANOVA with Tukey *post hoc* test. **F,** Schematic of a model which integrates the impact on centrosome/spindle and chromosomal missegregation observed following MEAP treatment. Scale bars, 10 μm.

To determine whether MEAP-induced centrosome hyperactivation is mediated through the NEDD9–STAT3 signaling axis, we compared MEAP’s effects in ALDH^+^ cells expressing control vectors, WT STAT3, or a constitutively active STAT3 mutant (STAT3-CA; Fig. 7A). We observed that both NEDD9 knockdown (shNEDD9) and STAT3-CA expression—but not WT STAT3, abolished MEAP-induced centrosome MT nucleation (Fig. 7B). Likewise, MEAP also induced increased γ-tubulin levels at the centrosomes in a STAT3-dependent manner (Fig. 7C). These findings collectively show that MEAP can selectively induce centrosome activation in ALDH^+^ cells by perturbing the NEDD9–STAT3 pathway. Although, MEAP did not significantly affect the overall rate of centrosome amplification, it markedly increased the ratio of multipolar to pseudobipolar spindles from 0.4:1 to 4:1 (Fig. 7D). These effects were specific to ALDH^+^ cells and were abolished in cells overexpressing constitutively active STAT3 (STAT3-CA) or expressing NEDD9 shRNA (shNEDD9R), supporting the mechanism of selective inhibition through the NEDD9–STAT3 axis. Notably, the subset (∼30%) of supernumerary centrosomes in control cells that lacked spindle-forming capacity was absent following MEAP treatment, mirroring effects observed with NEDD9 depletion (Fig. 7A). Pseudobipolar divisions in centrosome-amplified cells are known to promote chromosome missegregation, driving CIN. MEAP treatment reduced the frequency of missegregated chromosomes nearly threefolds (from ∼19% to ∼6%; Fig. 7E). This reduction was specific to ALDH^+^ cells and was not observed in ALDH^−^ cells or in ALDH^+^ cells expressing shNEDD9 or STAT3-CA, confirming that CIN suppression results from selective inhibition of the NEDD9–STAT3 pathway.

Together, these findings support the notion that MEAP’s selectivity for ALDH^+^ cells is facilitated by NEDD9–STAT3 interaction. Moreover, MEAP prevents centrosome-amplified ALDH^+^ cells from forming pseudobipolar spindles, redirecting their division fate from CIN-prone mitoses toward mitotic failure (Fig. 7F).

## Discussion

Abnormal cytokinesis resulting from centrosome defects is a common occurrence across many cancer types, contributing to CIN, tumor heterogeneity, and metastatic progression (39). In our previous work (2), we demonstrated that subpopulations of ATC cells with elevated expression of the stem cell marker ALDH1A1 exhibit enhanced centrosome clustering and efficiently propagate CIN due to their tolerance of centrosome amplification and micronuclei. Centrosome amplification and genomic instability is a hallmark of numerous advanced cancers, including ATC, one of the most aggressive and treatment-resistant malignancies. Although multimodal treatment and the introduction of BRAF-directed therapy with dabrafenib plus trametinib, alone or combined with immune checkpoint blockade, have improved outcomes for selected patients with BRAF V600E-mutant disease, durable disease control remains uncommon, and resistance is nearly universal (40–42). In this context, MEAP, which selectively disrupts centrosome clustering in cancer cells with supernumerary centrosomes, thereby inducing multipolar spindle formation and impairing migratory and invasive capacities, represents a mechanistically distinct, biology-driven strategy that could complement existing targeted and immunotherapeutic approaches in ATC. Developing strategies that selectively induce multipolar spindle formation in aggressive ALDH^+^ ATC cells by exploiting their supernumerary centrosomes represents a promising therapeutic approach to disrupt ATC progression (43).

In this study, we identified MEAP, a derivative of reversine capable of inducing maturation of supernumerary centrosomes that would otherwise remain inactive. We further show that MEAP enhances spindle multipolarity by increasing the proportion of centrosomes able to form robust spindle poles during mitosis. As pseudobipolar spindle formation typically relies on the partial inactivation of centrosomes, our findings suggest that MEAP disrupts this process by promoting centrosome maturation, thereby preventing spindle pole resolution and driving mitotic catastrophe. These results align with prior work demonstrating that mitotic catastrophe can be induced through centrosome activation by inhibiting CPAP–tubulin interactions (13). Importantly, we also show that in ATC, this centrosome activation strategy is particularly effective against ALDH^+^ cells, which are enriched in PCM-deficient (“inactive”) centrosomes during interphase and thus are highly sensitive to MEAP’s PCM-inducing effects. Although MEAP triggers nonselective apoptosis and senescence across ALDH subsets, centrosome declustering and G2/M accumulation occur preferentially in ALDH^+^ cells. This suggests that ALDH^+^ tumor cells are uniquely vulnerable to multipolar mitosis due to preexisting supernumerary centrosomes, whereas surviving ALDH^−^ cells activate compensatory DNA damage and senescence pathways at similar rates. Although our findings support preferential G2/M arrest and spindle multipolarity in ALDH^+^ cells, we lack direct mechanistic evidence that these cells undergo *bona fide* mitotic catastrophe rather than apoptosis or senescence. Future studies incorporating time-lapse imaging, caspase inhibition (e.g., Z-VAD), and senescence-associated secretory phenotype analyses will be required to dissect the fate of G2/M-arrested ALDH^+^ cells following MEAP treatment. Consequently, MEAP exhibits selective activity against ALDH^+^ cells, leading to reduced stemness within this aggressive cell population.

NEDD9 overexpression is observed in ALDH^+^ cells and distinguishes centrosome regulation between ALDH^+^ and ALDH^−^ populations (2). This suggests that targeting NEDD9-interacting partners may hold therapeutic potential for advanced ATC, as direct targeting of the multifunctional adapter protein NEDD9 remains challenging. Interestingly, MEAP is a distinct inhibitor with activity against both Aurora-A and FAK. Notably, NEDD9 depletion destabilizes Aurora-A kinase and enhances the efficacy of Aurora-A inhibitors (44). However, the Aurora-A selective inhibitor alisertib and the FAK inhibitor PF-573228 reproduced MEAP’s activity only partially, indicating that MEAP exerts superior efficacy through combined or additional mechanisms. Given Aurora-A’s central role in centrosome regulation (45–47) and its functional connection to NEDD9 (15, 16, 38, 48–55), Aurora-A inhibition is likely to be one of the drivers of centrosome-related effects in this study.

Moreover, the centrosome-activating effect of MEAP may provide a mechanistic explanation for the synergy reported earlier with Aurora-A inhibition and MT-targeting agents in ATC (56). Specifically, MEAP-induced enhancement of spindle multipolarity would be expected to potentiate the mitotic catastrophe triggered by paclitaxel-mediated MT stabilization in ATC cells.

Our study also demonstrated that MEAP activity is dependent on the NEDD9/STAT3 axis. Consistently, shNEDD9 and MEAP reduced cytoplasmic but not nuclear pSTAT3-Y705. Combining shNEDD9 or MEAP with overexpression of STAT3 or constitutively active STAT3 (STAT3-CA) showed that STAT3 activation functions downstream of the NEDD9 interactome to antagonize centrosome activation. These results indicate that maintaining precise pSTAT3 levels is critical for asymmetric centrosome configuration in ALDH^+^ ATC cells, a feature shared with other stem-like cells (57).

Our centrosome activation strategy represents a mechanistically distinct approach within the growing landscape of centrosome-targeting therapeutics. Whereas clustering inhibitors (HSET/KIFC1) and CPAP–tubulin antagonists suppress multipolarity by reducing pole number or promoting centrosome inactivation, MEAP exploits a complementary vulnerability by restoring PCM function to dormant supernumerary centrosomes. This orthogonal mechanism may offer synergistic potential when combined with MT-targeting agents, where MEAP-induced multipolarity could potentiate paclitaxel-mediated mitotic catastrophe through unresolved spindle tension and chromosome missegregation. Given precedent for taxane synergy in ATC and MEAP’s demonstrated *in vivo* activity, combination studies warrant investigation as a strategy to maximize mitotic failure in ALDH^+^ cancers with centrosome amplification.

This NEDD9–STAT3 interaction is consistent with previous findings in ovarian cancer, in which Nedd9^+^/^+^ cells exhibited higher pSTAT3-Y705 levels and NEDD9 expression correlated with ALDH1A1/ALDH1A2 (58). This aligns with studies showing that abnormal STAT3 activation occurs in multiple cancers and is associated with cancer cell stemness (59). STAT3 has also been reported to regulate centrosome clustering in breast cancer models (43). Together, these observations suggest that the NEDD9–STAT3 axis may have broad relevance for regulating centrosome dynamics and stem-like states across diverse cancer types.

In summary, MEAP targeting via the NEDD9–STAT3 axis may represent a broadly applicable strategy for combating advanced cancers with stem-like features and centrosome amplification.

## Supplementary Material

Figure S1 MEAP exhibits superior multikinase inhibition compared to reversine

Table S1 Impact of analogues on the colony forming abilities and proliferation of anaplastic thyroid carcinoma cells.

## Data Availability

All data are found within the article, supplemental files, or are available from authors upon request.
