# Supplementary material for: ALDH+ Anaplastic Thyroid Cancer Cells Show Vulnerability to a Pharmacologic Inducer of Centrosome Declustering
Source: Cancer Res Commun. 2026 May 19;6(5):1151–67. doi: 10.1158/2767-9764.CRC-25-0807 (PMC13184942; doi:10.1158/2767-9764.CRC-25-0807)
Supplement: Figure S1 — MEAP exhibits superior multikinase inhibition compared to reversine [file crc-25-0807_figure_s1_suppsf1.docx]

**
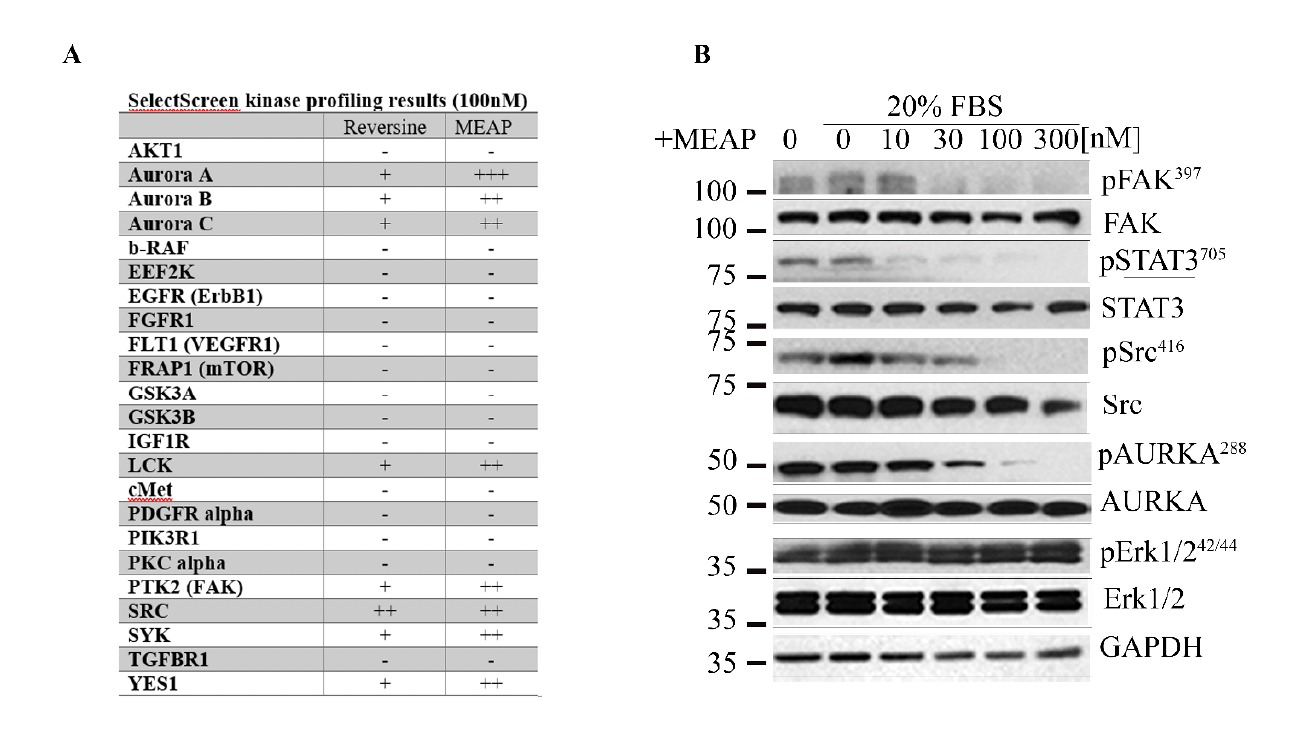
**

**Figure S1: MEAP exhibits superior multikinase inhibition compared to reversine**

**A.** In vitro kinase profiling (SelectScreen™, Thermo Fisher Scientific) shows MEAP inhibits NEDD9-interactors Aurora kinase A (AURKA) and FAK with higher potency than reversine. Inhibition scale: – (0–50%), + (51–70%), ++ (71–80%), +++ (81–100%). **B.** Western blots demonstrate dose-dependent kinase inhibition in ALDH+ cells after 1 h MEAP treatment.
