# Supplementary material for: ALDH+ Anaplastic Thyroid Cancer Cells Show Vulnerability to a Pharmacologic Inducer of Centrosome Declustering
Source: Cancer Res Commun. 2026 May 19;6(5):1151–67. doi: 10.1158/2767-9764.CRC-25-0807 (PMC13184942; doi:10.1158/2767-9764.CRC-25-0807)
Supplement: Table S1 — Impact of analogues on the colony forming abilities and proliferation of anaplastic thyroid carcinoma cells. [file crc-25-0807_table_s1_suppst1.docx]

**Table S1:** Impact of analogues on the colony forming abilities and proliferation of anaplastic thyroid carcinoma cells.

**
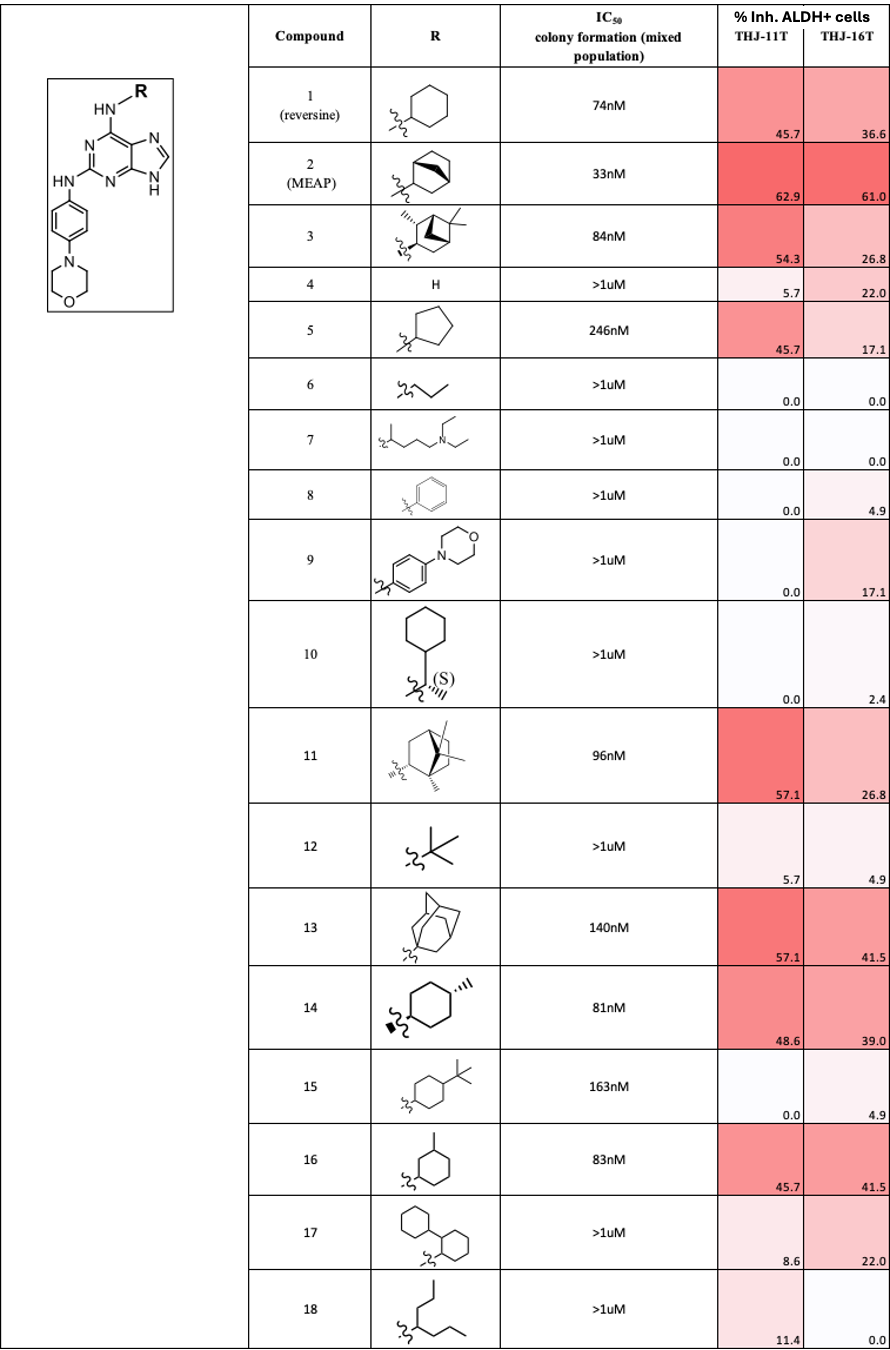
**
